# Quantitative and qualitative analysis of dental clinics waste in Zabol city, Iran

**DOI:** 10.1016/j.dib.2018.06.041

**Published:** 2018-06-22

**Authors:** Gholam Reza Ebrahimzadeh, Samira Norzaee, Babak Djahed, Ebrahim Enayat, Yadolah Fakhri, Mahmoud Taghavi

**Affiliations:** a Department of Environmental Health Engineering, Zabol University of Medical Sciences, Zabol, Iran; b Department of Environmental Health Engineering, Iranshahr University of Medical Sciences, Iranshahr, Iran; c Department of Environmental Health Engineering, Student Research Committee, School of Public Health, Shahid Beheshti University of Medical Sciences, Tehran, Iran; d Department of Environmental Health Engineering, Social Determinants of Health Research Center, Gonabad University of Medical Sciences, Gonabad, Iran

**Keywords:** Dentistry waste, Dental clinics, Waste composition, Infectious waste, Toxic waste

## Abstract

Dental clinics are one of the sources of waste production that are important due to producing infectious and potentially infectious waste, chemical and pharmaceutical waste, and toxic waste. Therefore, this study aimed to analyze dental clinics in Zabol quantitatively and qualitatively. This descriptive cross-sectional study was conducted in 2014 on waste produced in dental clinics in Zabol. Sampling of 25 dental clinics was performed three times per week. At the end of the working day, the samples were transferred to a suitable site and weighed carefully after separation of the components. Data were analyzed using descriptive statistics and Excel software. 5457 kg of waste is annually produced in the dental clinics of Zabol that the amount of infectious and potentially infectious waste, household-like waste, chemical and pharmaceutical waste, and toxic wastes are approximately 48.08, 43.75, 7.82 and 0.35%, respectively. Given that proper management of waste produced is not performed in dental clinics in Zabol, special attention to waste produced in this sector through programs of reduction in source, separation and recycling can reduce the waste volume significantly.

**Specifications Table**

**Table:** 

Subject area	Environmental science
More specific subject area	Waste management,
Type of data	Tables, Figure
Data collection method	Sampling of 25 clinics was conducted on three occasions and on Monday, Tuesday and Wednesday. At the end of the workday, samples were transferred to a suitable site and then weighed. Weighing of samples was such that first waste was separated into components and weighed using scale model SP-400. To estimate waste produced per person per year, the number of workdays in 2014 was determined according to the country official calendar equal to 286 days.
Data format	Raw/Analyzed
Experimental factors	Composition of dental waste/Weight of dental waste components
Experimental features	Waste produced per person per year/Per capita waste production was determined
Data source location	Zabol, Sistan and Baluchistan Province, Iran
Data accessibility	The data are available in this article

**Value of the data**

• The data presented in this article present a detailed description of dental waste produced in private dental clinics.
• The data can be useful for managers of municipalities to select best methods to manage dental waste and supply necessary equipment and facilities.
• Quantitative and qualitative data on dental waste could assist decision makers on source reduction, separation and recycling programs.

## 1 Data

**Fig. 1:**
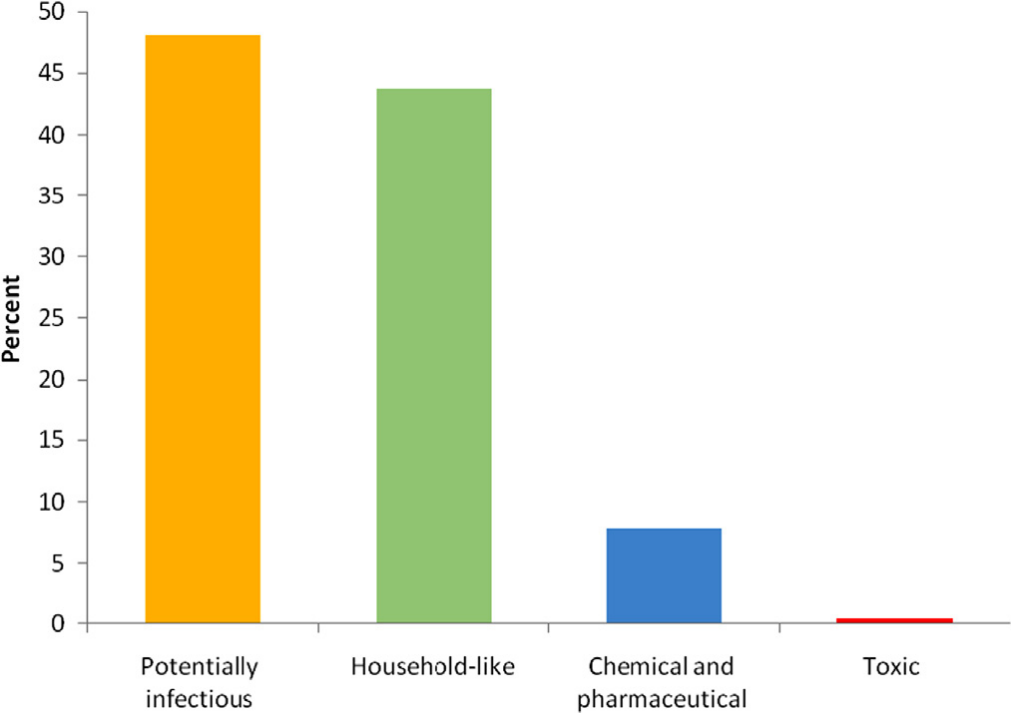
Percentage of potentially infectious, household-like, chemical, pharmaceutical and toxic waste produced in the dental clinics in Zabol.

Healthcare waste is a highly dangerous waste group requiring special attention. According to the definition of the World Health Organization, this waste contains substances resulting from health care activities on humans and animals and infectious agents. Approximately 75–90% of the waste produced in health care centers is non- hazardous or public solid waste, but 10–25% of the remaining waste is hazardous. This group of waste has health and environmental hazards due to containing infectious agents, sharp objects, pathological waste, hazardous chemicals or pharmaceutical waste and or having genotoxic and radioactive effects [1], [2], [3], [4]. This paper presents data supporting quantitative and qualitative analysis of dental clinics׳ waste in Zabol city, Iran. According to the results of this study, the total amount of waste produced in the private dental clinics in Zabol is 5456.9 kg/y of which 2623.53 kg per year is related to potentially infectious waste, and 2387.35 kg is related to household-like waste. The amount of chemical, pharmaceutical and toxic waste is 426.71 and 19.3 kg, respectively. Fig. 1 shows the percentage of the waste produced in private dental clinics in Zabol. As shown, potentially infectious waste constitutes 47.55% of waste. Table 1, Table 2, Table 3, Table 4 depict the amount and percentage of potentially infectious waste, household-like waste, chemical and pharmaceutical waste and toxic waste produced in the dental clinics of Zabol.

**Table 1:** The amount and percentage of different components of the potentially infectious waste produced in the dental clinics of Zabol.

**The type of waste produced**	**The amount of waste produced (kg/y)**	**Percentage**
Tooth extracted	22.47	0.86
Latex gloves	881.86	33.61
Mouth stick	168.48	6.42
Blood-contaminated cotton	35.14	1.34
plastic syringe	221.46	8.44
Nylon gloves	172.8	6.59
Blood-contaminated paper towel	260.59	9.93
Suction tip	181.73	6.93
Saliva-contaminated paper towel	104.03	3.97
Needles and sharp objects	217.15	8.28
Saliva-contaminated dental roll	104.03	3.97
Dental mirrors	70.56	2.69
Blood-contaminated dental roll	23.13	0.88
Saliva-contaminated cotton	24.15	0.92
Blood-contaminated bandage	68.04	2.59
Dental spatula	8.93	0.34
Saliva-contaminated bandage	54.38	2.07
Paper cone	4.6	0.18
Total	2623.53	100.00

**Table 2:** The amount and percentage of different components of the household-like waste produced in the dental clinics of Zabol.

**The type of waste produced**	**The amount of waste produced (kg/y)**	**Percentage**
Nylon	659.76	27.64
Dental disposable tray	457	19.14
Paper and newspaper	294.04	12.32
Molding plaster	178.61	7.48
Mouth mask	148.08	6.20
Glass	127.75	5.35
Tea waste	95.5	4.00
Paper cup	86.57	3.63
Empty amalgam capsule	73.68	3.09
Metal	64.68	2.71
Hat and apron	37.6	1.57
Other	164.08	6.87
Total	2387.35	100.00

**Table 3:** The amount and percentage of different components of the chemical and pharmaceutical waste produced in the dental clinics of Zabol.

**The type of waste produced**	**The amount of waste produced (kg/y)**	**Percentage**
Molding plaster	42.04	9.85
Gutta-percha	15.83	3.71
X-ray film	15.18	3.56
Consumed ampoule	353.66	82.88
Total	426.71	100.00

**Table 4:** The amount and percentage of different components of the toxic waste produced in the dental clinics of Zabol.

**The type of waste produced**	**The amount of waste produced (kg/y)**	**Percentage**
Lead cover of X-ray film	14.98	77.62
Amalgam	4.32	22.38
Total	19.3	100.00

## 2 Experimental design, materials and methods

### 2.1 Study area description

**Fig. 2:**
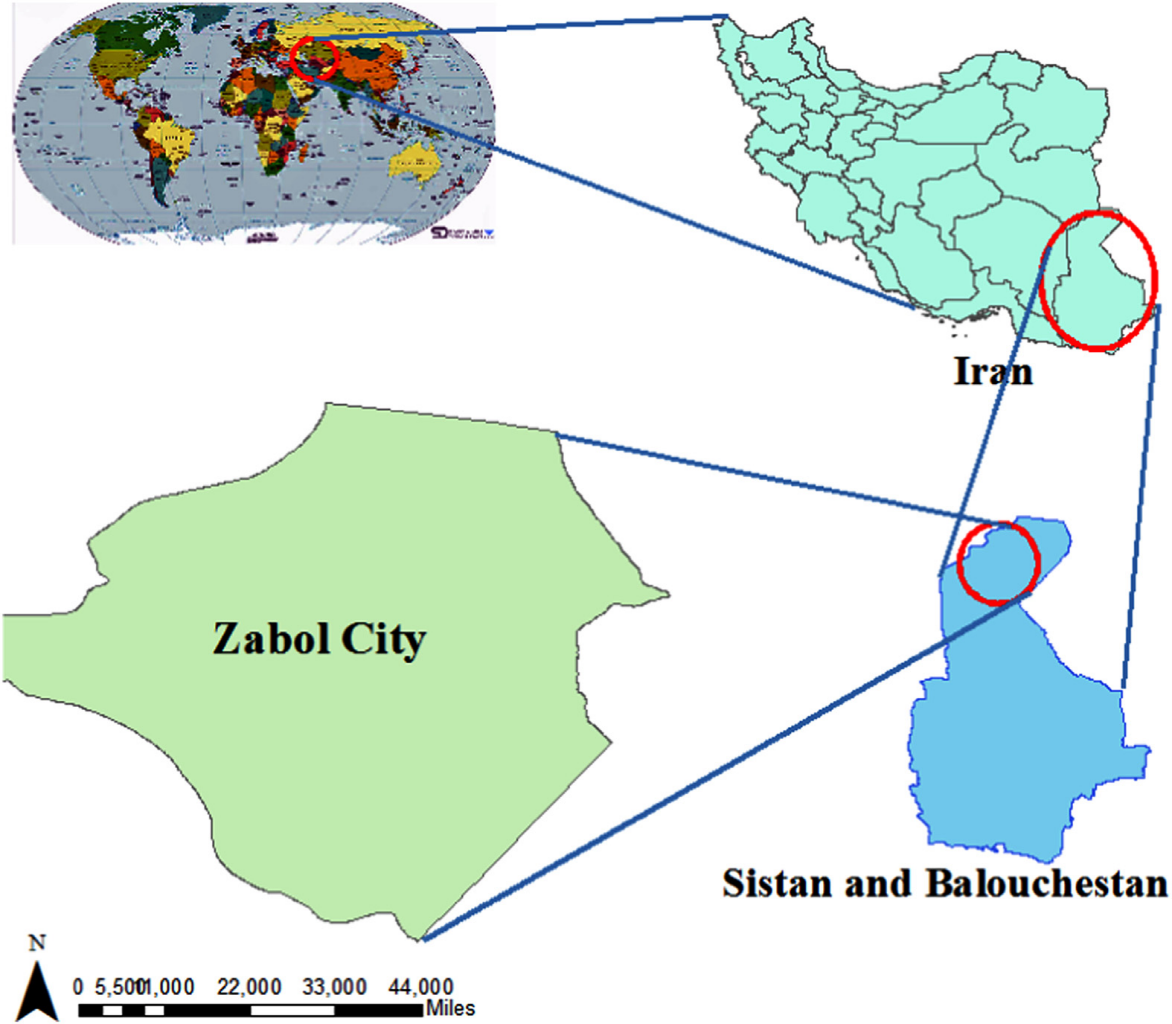
The location of the study area, Zabol city, Sistan and Baluchistan Province, Iran.

Zabol city is the capital of Zabol County, Sistan and Baluchestan Province (Fig. 2), which lies on the border with Afghanistan, and has a total area of approximately 344 km^2^. Population of Zabol was 137,722 in 2011.

### 2.2 Sample collection and analytical procedures

Zabol has 31 private dental clinics of which 25 clinics cooperated for conducting the study. Sampling was performed every week with no public holiday. Sampling of 25 clinics was conducted on three occasions and on Monday, Tuesday and Wednesday. At the end of the workday, samples were transferred to a suitable site and then weighed. Weighing of samples was such that first, waste was separated into components and weighed using scale model SP-400. The number of patients referred to each dental clinic was determined to investigate the daily per capita waste production per patient. To estimate waste produced per person per year, the number of workdays in 2014 was determined according to the country official calendar equal to 286 days. Given that all 31 private dental clinics were not willing to participate in this study, measurements were multiplied by a factor of 1.4 to estimate the total waste produced in the city.
